# H_2_O_2_ selectively damages the binuclear iron-sulfur cluster N1b of respiratory complex I

**DOI:** 10.1038/s41598-023-34821-5

**Published:** 2023-05-11

**Authors:** Lisa Strotmann, Caroline Harter, Tatjana Gerasimova, Kevin Ritter, Henning J. Jessen, Daniel Wohlwend, Thorsten Friedrich

**Affiliations:** 1 Institut für Biochemie, Albert-Ludwigs-Universität Freiburg, Albertstr. 21, 79104 Freiburg, Germany; 2 Institut für Organische Chemie, Albert-Ludwigs-Universität Freiburg, Albertstr. 21, 79104 Freiburg, Germany

**Keywords:** Bioinorganic chemistry, Enzyme mechanisms, Structural biology, Bioenergetics, Biocatalysis, Enzyme mechanisms, Enzymes

## Abstract

NADH:ubiquinone oxidoreductase, respiratory complex I, plays a major role in cellular energy metabolism by coupling electron transfer with proton translocation. Electron transfer is catalyzed by a flavin mononucleotide and a series of iron-sulfur (Fe/S) clusters. As a by-product of the reaction, the reduced flavin generates reactive oxygen species (ROS). It was suggested that the ROS generated by the respiratory chain in general could damage the Fe/S clusters of the complex. Here, we show that the binuclear Fe/S cluster N1b is specifically damaged by H_2_O_2_, however, only at high concentrations. But under the same conditions, the activity of the complex is hardly affected, since N1b can be easily bypassed during electron transfer.

## Introduction

Energy-converting NADH:ubiquinone oxidoreductase, respiratory complex I, plays an important role in cellular bioenergetics by coupling NADH oxidation and ubiquinone (Q) reduction with the translocation of protons across the membrane^1–6^. It consists of a peripheral arm catalyzing electron transfer and a membrane arm responsible for proton translocation. The two arms are arranged nearly perpendicular to each other resulting in an L-shaped structure of the complex. Mitochondrial complex I consists of 45 subunits including 14 core subunits that are found in all species containing an energy-converting NADH:Q oxidoreductase^7,8^. The three-dimensional structure of the core subunits of complex I is conserved from bacteria to mammals^9,10^. The bacterial complex from *Escherichia coli* is composed of 13 different subunits that are named NuoA to NuoN, with two of them being fused to the single subunit NuoCD^11^. They are encoded by the *nuo*-genes and add up to a molecular mass of approximately 530 kDa^12^.

NADH is oxidized at the tip of the peripheral arm by hydride transfer to the primary electron acceptor flavin mononucleotide (FMN)^13^. From here, electrons are transferred over a distance of approximately 100 Å via a series of seven iron-sulfur (Fe/S) clusters towards the membrane, where Q is reduced and protonated in a specific binding cavity that is made up of subunits of the peripheral and the membrane arm^1–6^. A Q species is thought to move from a high-energy to a low-energy binding site inside the cavity, causing electrostatic and conformational changes that drive proton translocation in the membrane arm^2,14–16^. The membrane arm contains four putative proton pathways that are connected to each other and to the Q cavity by a central axis of charged residues. It was proposed that the movement of the Q species in its cavity induces the propagation of an ‘electric’ wave that moves forth and back through the membrane arm triggering proton translocation^16^. Alternatively, it has been suggested that the binding of quinone leads to a transition from an ‘open’ to a ‘closed’ state^10^. Quinone reduction results in a re-distribution of protons in the membrane arm, which in turn leads to a proton release to the cytoplasm exclusively in NuoL^10^.

NADH oxidation by complex I is associated with the production of reactive oxygen species (ROS) such as superoxide and hydrogen peroxide^17–20^ contributing to cellular stress^21^. It is generally accepted that ROS generated by complex I originate at the reduced FMN^17–20^. About 0.1–2% of the oxidized NADH lead to ROS production in vitro^22–25^. ROS not only contribute to oxidative damage such as lipid peroxidation, protein degradation and DNA oxidation, but also represent essential redox signals^26–29^.

The ROS-producing FMN cofactor is located in the immediate vicinity of the Fe/S clusters of respiratory complex I. It is well known that solvent exposed Fe/S clusters are prone to oxidative damage^30,31^. The structures of complex I from different organisms show that its Fe/S clusters are mostly shielded from the solvent and, thus, should be protected from degradation by ROS^7–10,32–36^. Nevertheless, it was proposed that an enhanced ROS production by complex I and the respiratory chain in general may result in damage to the Fe/S clusters of complex I^37^. Here, we used the *E. coli* complex I to test this proposal. By representing a structural minimal form of the mitochondrial complex I, the one from *E. coli* lacks the additional accessory subunits surrounding the catalytic core. Therefore, the Fe/S clusters of *E. coli* complex I might be more susceptible to oxidative damage than their homologues in mitochondrial complex I. Since it is known that *E. coli* complex I produces ROS mainly in the form of H_2_O_2_^38,39^, we assayed the influence of H_2_O_2_ on complex I activity and its Fe/S cluster composition. It turned out that millimolar H_2_O_2_ concentrations are needed to inhibit NADH oxidase activity. While the NADH:decyl-ubiquinone activity of the isolated complex remains unchanged in the presence of 1 mM H_2_O_2_, we directly demonstrate using EPR spectroscopy that treatment with 1 mM H_2_O_2_ results in a selective loss of the Fe/S cluster N1b on subunit NuoG.

## Results

### Inhibition of complex I by H_2_O_2_

Due to the activity of cellular catalases and peroxidases, the H_2_O_2_ concentration in the *E. coli* cytoplasm is in the low nanomolar range^40,41^. However, the addition of exogenous H_2_O_2_ can increase the interim intracellular concentration to the micromolar range^42^. Thus, the effect of micromolar H_2_O_2_ concentrations on complex I-mediated NADH oxidation was measured initially with the protein in membranes. Cytoplasmic membranes of strain BW25113Δ*ndh nuo*:*npt*II_FRT/pBAD*nuo*_*His*_ were obtained by differential centrifugation. Due to the lack of the alternative NADH dehydrogenase (*ndh*) and the disruption of the chromosomal *nuo*-operon, all NADH-derived activities of membranes from this strain exclusively reflect activities of wild-type complex I encoded on the plasmid.

**Figure 1:**
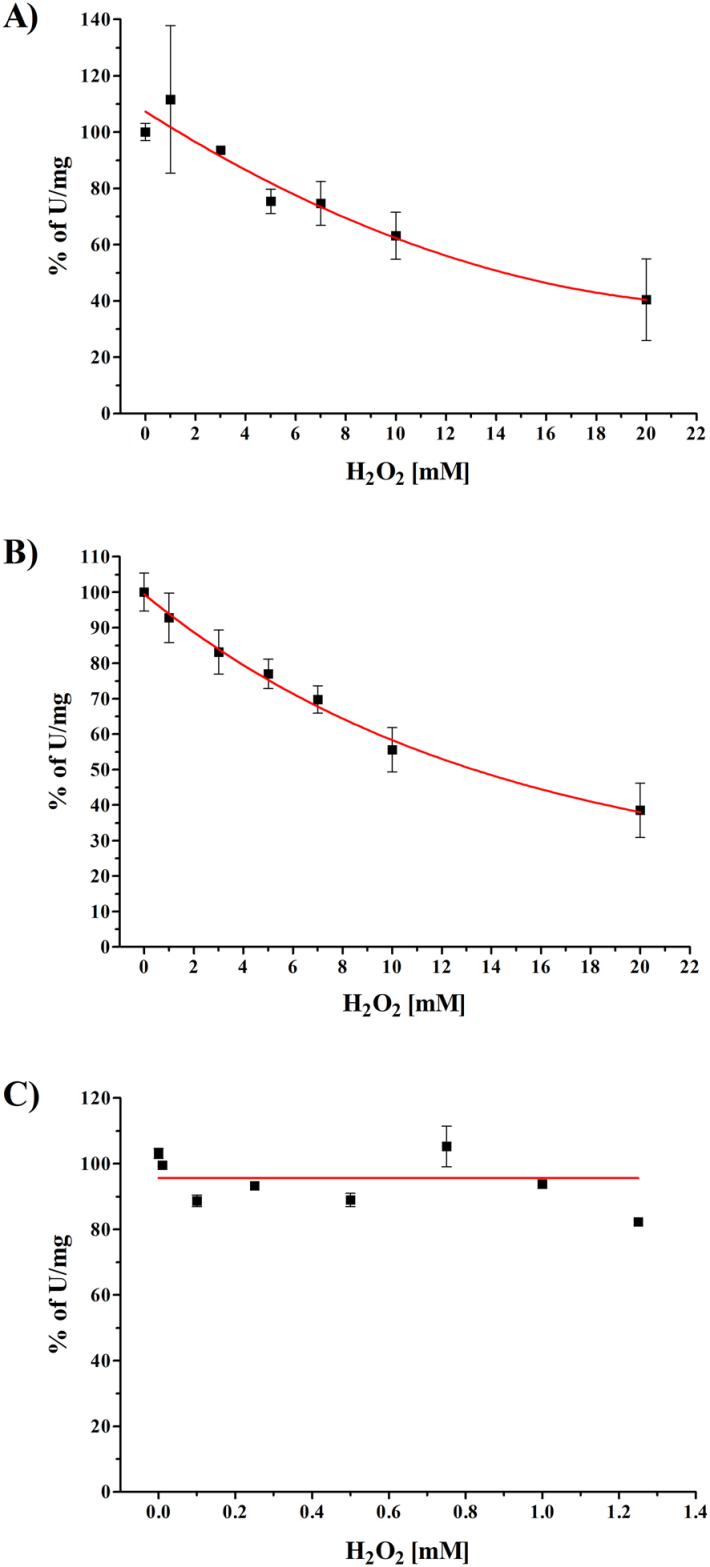
Inhibition of complex I by H_2_O_2_. (**A**) NADH/ferricyanide oxidoreductase activity of membranes from strain BW25113Δ*ndh nuo*:*npt*II_FRT/pBAD*nuo*_*His*_. 100% activity corresponds to 1.4 U mg^−1^. (**B**) NADH oxidase activity of membranes from strain BW25113Δ*ndh nuo*:*npt*II_FRT/pBAD*nuo*_*His*_. 100% activity corresponds to 0.27 U mg^−1^. (**C**) NADH:decyl-ubiquinone oxidoreductase activity of isolated complex I. 100% activity corresponds to 21.9 U mg^−1^. The red lines through the data points are included only as a guide. Each data point is the average of three technical replicates from two biological samples. The bars represent the SEM at each data point.

The NADH/ferricyanide oxidoreductase activity of complex I is catalyzed by the FMN bound to NuoF and does not involve the participation of Fe/S clusters. In addition, this activity is not coupled with proton translocation. It turned out that micromolar H_2_O_2_ concentrations had no effect on this activity. Only millimolar concentrations exerted a significant effect on the NADH/ferricyanide oxidoreductase activity (Fig. 1A). Titration with up to 20 mM H_2_O_2_ resulted in 55% inhibition of the activity with an apparent IC_50_ of 14.4 mM.

To assay the effect of H_2_O_2_ on the physiological activity of complex I including electron transfer via the Fe/S clusters to Q, its influence on the NADH oxidase activity was determined (Fig. 1B). As already observed for the NADH/ferricyanide oxidoreductase activity, H_2_O_2_ inhibits the NADH oxidase activity only in the millimolar range. Titration with up to 20 mM H_2_O_2_ resulted in an approximately 55% inhibition of the activity with an apparent IC_50_ of 13.5 mM. The similarity of both inhibition curves suggests a non-specific effect of H_2_O_2_ on complex I in the membrane.

To determine whether the inhibition is reversible, an untreated aliquot of membranes and an aliquot treated with 20 mM H_2_O_2_ were centrifuged three times and each was re-suspended in buffer A without H_2_O_2_. The NADH/ferricyanide and NADH oxidase activity of the treated sample was 50 ± 5% of that of the untreated in both cases. This suggests that the inhibition by H_2_O_2_ is irreversible.

To specifically investigate the effect of H_2_O_2_ on complex I, the protein was isolated in the presence of the detergent LMNG (see Supplementary Fig. S1) and the inhibition of the NADH:decyl-Q oxidoreductase activity of the preparation by H_2_O_2_ was measured. In the range up of to 1.25 mM H_2_O_2_ no effect on complex I activity was detectable (Fig. 1C). An addition of 20 mM H_2_O_2_ resulted in 65% inhibition of the activity. In summary, our experiments demonstrate that at concentrations observed under physiological conditions, H_2_O_2_ has no effect on the activity of complex I.

### Oxidation of complex I Fe/S clusters by H_2_O_2_

To determine whether H_2_O_2_ is nevertheless capable of damaging the Fe/S clusters of the complex, a preparation was split into aliquots and samples were either treated with buffer or with an equal volume H_2_O_2_ at various concentrations. The samples were reduced by a 2000 fold molar excess of NADH, incubated with H_2_O_2_ for one minute at ambient temperature and then frozen in a refrigerant solution at 150 K. EPR spectra were recorded at 40 K and 2 mW microwave power to detect the two binuclear Fe/S clusters of complex I, N1a and N1b. In addition, spectra were recorded at 13 K and 5 mW to detect the tetranuclear clusters N2, N3, and N4. The other Fe/S clusters of the complex are not detectable by EPR^43^. The sample supplied with only buffer was used as reference. Samples that were first treated with H_2_O_2_ and then reduced by NADH resulted in similar EPR spectra. Incubation of the samples with H_2_O_2_ for 30 min either before or after reduction by NADH did not result in spectral changes.

**Figure 2:**
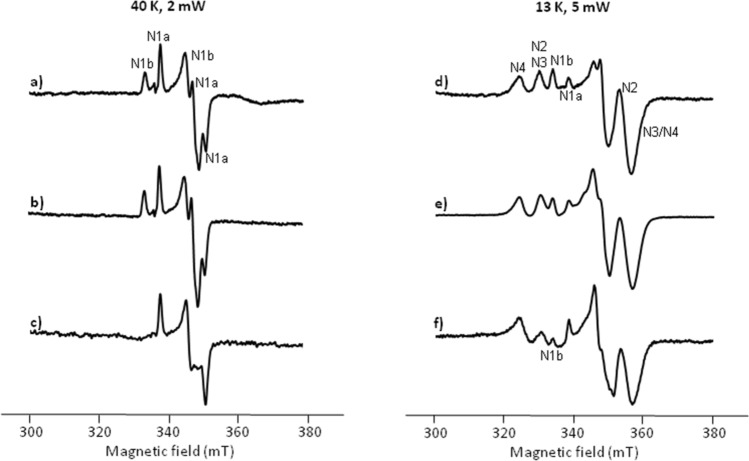
EPR spectra of isolated *E. coli* complex I at various H_2_O_2_ concentrations. Spectra were recorded from an untreated sample (**a**,**d**), an aliquot incubated with 100 µM (**b**,**e**) and 1 mM (**c**,**f**) H_2_O_2_. Spectra were recorded at 40 K and 2 mW microwave power (left row, **a**–**c**) to detect the binuclear Fe/S clusters and at 13 K and 5 mW power (right row, **d**–**f**) to additionally detect the tetranuclear Fe/S clusters. Individual signals are assigned to the distinct Fe/S clusters according to^59^. The central g-region around g = 1.94 is disturbed in (**f**) due to the lack of g_⊥_ of N1b at 1.94.

The spectrum of the reference sample obtained at 40 K and 2 mW microwave power showed the presence of the binuclear clusters N1a (g_x,y,z_ = 1.92, 1.94, and 2.00) and N1b (g_//,⊥_ = 2.03 and 1.94; Fig. 2a). The signals of the tetranuclear Fe/S clusters N2 (g_//,⊥_ = 1.91 and 2.05), N3 (g_x,y,z_ = 1.88, 1.92, and 2.04), and N4 (g_x,y,z_ = 1.89, 1.93, and 2.09) were present in the spectrum recorded at 13 K and 5 mW microwave power in addition to the signals of the binuclear clusters (Fig. 2d).

EPR spectra of the samples that were incubated with up to 100 µM H_2_O_2_ did not show any significant spectral change (Fig. 2b,e). However, the sample treated with 1 mM H_2_O_2_ clearly showed a drastic and specific loss of cluster N1b (Fig. 2c,f). The difference of the spectrum of the untreated sample obtained at 40 K minus that of the sample treated with 1 mM H_2_O_2_ clearly shows the signals of cluster N1b (see Supplementary Fig. S2). Thus, the proportion of N1a has not changed, as its signal would otherwise also be detectable in the difference spectrum. However, a complete oxidation of N1b cannot be deduced from the difference spectrum. The loss of cluster N1b is also seen in the spectra recorded at 13 K and 5 mW microwave power (Fig. 2c,f). Here, a residual amount N1b is still detectable in the spectra recorded at 13 K (Fig. 2f) suggesting that the cluster is not completely damaged. Double integration of the N1b signal at g = 2.03 that has no overlap with other signals showed that 68% of N1b are damaged by 1 mM H_2_O_2_. However, the amount of the binuclear cluster N1a and of the tetranuclear clusters N2, N3 and N4 did not change even in the presence of 50 mM H_2_O_2_. Thus, 1 mM H_2_O_2_ leads to a specific damage of the binuclear cluster N1b, while even highest H_2_O_2_ concentrations in our assays have no effect on the other Fe/S clusters of complex I.

To distinguish whether cluster N1b is merely oxidized or 'over-oxidized' by H_2_O_2_, complex I was incubated for 5 min with 1 mM H_2_O_2_. Subsequently, the excess H_2_O_2_ was removed by repeated concentration and dilution. The sample was reduced with NADH and the EPR spectrum shows the lack of cluster N1b (see Supplementary Fig. S3). Thus, N1b is irreversibly damaged by H_2_O_2_ and not simply oxidized.

### Solvent channels to cluster N1b

**Figure 3:**
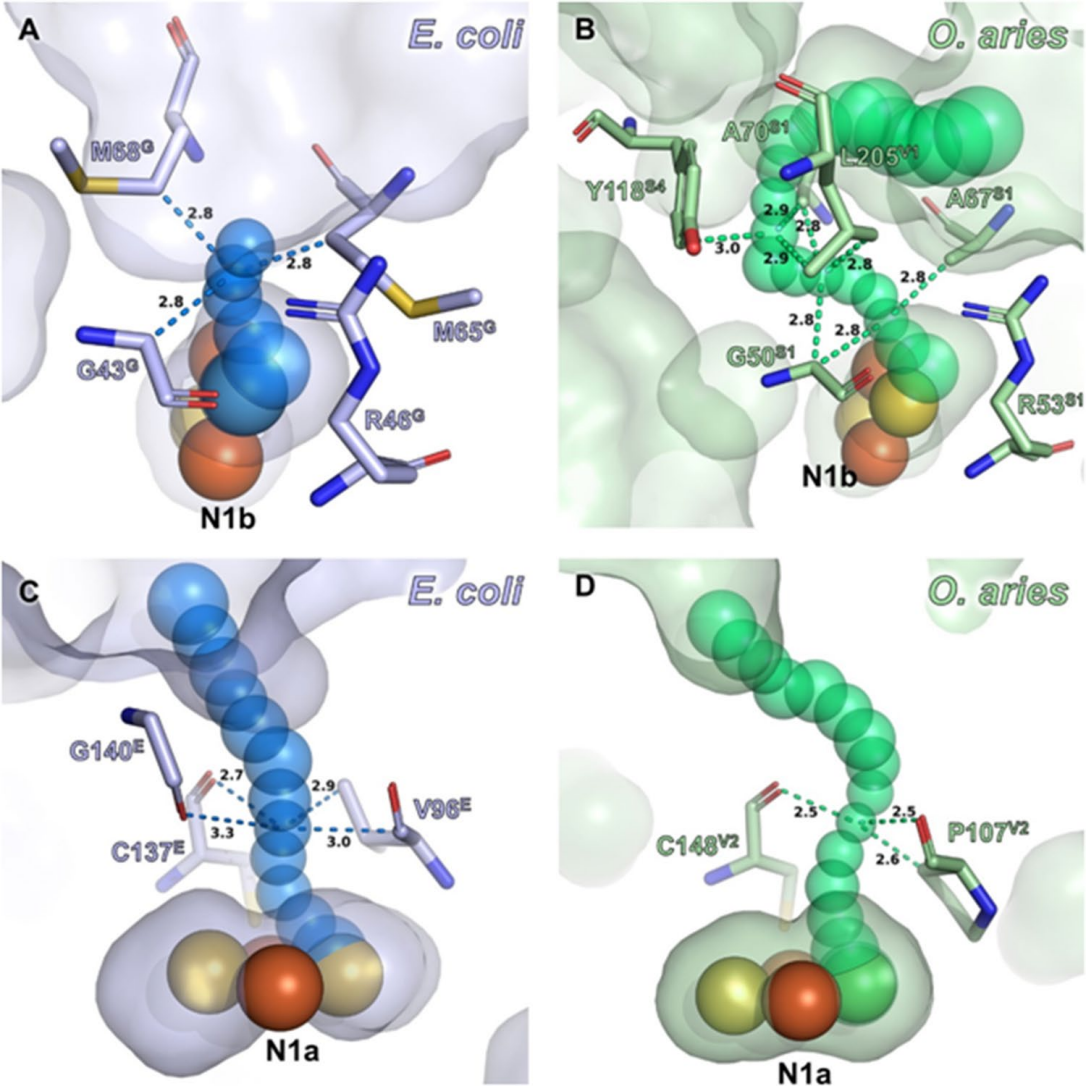
Surface accessibilities for H_2_O_2_ of clusters N1a and N1b in *E. coli* and *O. aries*. Surface channels were probed with CAVER 3.0.3 on PDB IDs 7AWT (*E. coli*) and 7ZD6 (*O. aries*). Distances of lining atoms to the channel centers at constrictions are provided and additionally indicated with dashes. (**A**,**B**) Solvent channels leading toward N1b in *E. coli* (**A**) and *O. aries* (**B**). The provided diameters are narrow, yet presumably sufficient to allow passage of H_2_O_2_ to N1b with 2.28 Å in *E. coli* and 2.26 Å in *O. aries*. Note that in *O. aries* complex I, a direct way to the cluster is blocked by R53 of NDUFS1. The linear distance of N1b to the solvent accessible surface amounts to 4.9 Å (*E. coli*) and 8.2 Å (*O. aries*), respectively (not indicated). (**C**,**D**) H_2_O_2_ accessibility of N1a is restricted with channel diameters of 1.98 Å (*E. coli*) and 1.90 Å (*O. aries*). The apolar atoms at the constrictions efficiently repel polar molecules. The minimal distance of N1a iron atoms to the surface is 7.3 Å (*E. coli*) and 9.3 Å (*O. aries*), respectively (not indicated).

The structure of the complex from various species shows that the Fe/S centers are not solvent exposed. To find out why N1b can be attacked by H_2_O_2_ nonetheless, we took a closer look at the cryo-EM structure of the peripheral arm of the *E. coli* complex that contains all Fe/S clusters^44^. N1b is coordinated by four cysteine residues of a typical [2Fe–2S] ferredoxin fold at the N-terminal part of NuoG (Fig. 3). This domain connects NuoE and NuoF with NuoG. Cluster N1b is localized in about 5 Å distance to the surface of NuoG but without direct solvent access (Fig. 3). We used the program CAVER to identify possible solvent channels that might lead from the protein surface to the cluster. A channel with a diameter of 2.28 Å leads from the solvent to the cluster (Fig. 3A). The channel is flanked by residues Gly45^G^, Arg46^G^ and Met67^G^ (the superscript refers to the name of the subunit of *E. coli* complex I). Mitochondrial complex I contains several accessory subunits, whose number depends on the particular organism. However, none of these accessory subunits further shields cluster N1b towards the solvent. Exemplarily, the environment of cluster N1b of the complex I from ovine mitochondria^36^ is shown in Fig. 3B. In mitochondrial complex I, N1b is positioned about 8.2 Å beneath the protein surface. Here, the channel has a slightly smaller diameter of 2.26 Å and extends over a longer distance in a U-shaped fashion (Fig. 3B). This is due to the presence of Leu225 from subunit NDUFV1, the homologue of *E. coli* NuoF, and Arg53 from NDUFS1, the homologue of *E. coli* NuoG, that both block the direct path to the cluster. In ovine complex I, the path is further gated by residues Gly50, Arg53, Ala67 and Ala70 from NDUFS1 and Tyr118 from subunit NDUFS4, an accessory subunit that is a structural homologue of an extra-domain of NuoG^44^. The solvent channels are sufficiently large in both organisms to allow the passage of H_2_O_2_ to the cluster. Some of the lining atoms are hydrophobic and hamper rapid passage, yet without blocking it. Thus, an addition of H_2_O_2_ would presumably also lead to a damage of N1b in mitochondrial complex I.

## Discussion

It has been previously reported that H_2_O_2_ has no effect on *E. coli* complex I activity^42^. However, no data were shown and it was only mentioned that 5 mM H_2_O_2_ do not diminish its activity. From this data it was concluded that H_2_O_2_ does not oxidize the Fe/S clusters of complex I^42^. Here, we show that incubation of *E. coli* membranes with 5 mM H_2_O_2_ leads to a small but significant inhibition of NADH oxidase activity, when complex I is overproduced. Half-maximal inhibition is achieved at 13.5 mM H_2_O_2_ (Fig. 1B). The inhibition of NADH/ferricyanide oxidoreductase activity showed a similar activity course with an IC_50_ of 14.4 mM H_2_O_2_ (Fig. 1A). However, incubation of free FMN and NADH with 20 mM H_2_O_2_ does not lead to their chemical modification (see Supplementary Fig. S4). From this, we propose that the decrease in NADH oxidase and NADH/ferricyanide oxidoreductase activities at high H_2_O_2_ concentrations (Fig. 1A,B) is most likely due to unspecific protein oxidation^45^.

**Figure 4:**
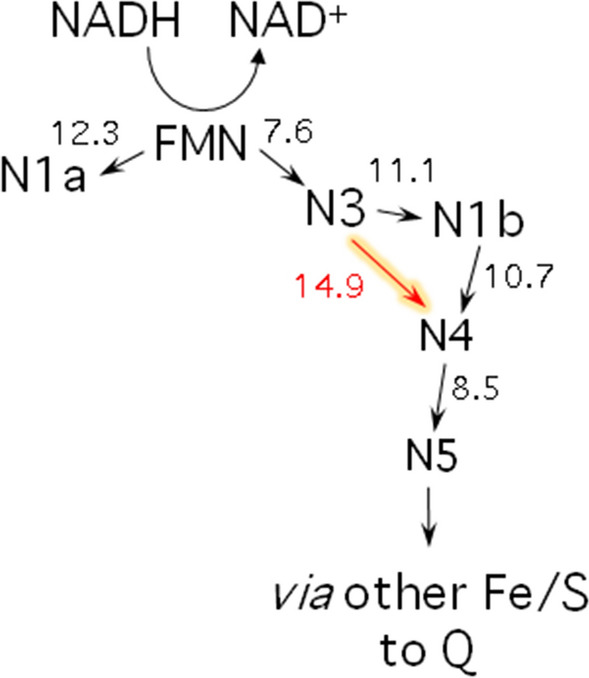
Scheme showing the arrangement of the Fe/S clusters in the electron input module of *E. coli* complex I. The relative positions of clusters N1a (NuoE), N3 (NuoF) and N1b, N4 and N5 (all NuoG) are shown. The arrows indicated possible electron transfer pathways. The edge-to-edge distances between the clusters in Å are indicated on the respective arrows. The lack of N1b can be bypassed by direct electron transfer from N3 to N4 (red; nomenclature according to^59^).

However, in contrast to the assumption that H_2_O_2_ has no effect on the Fe/S cluster of the complex^42^, we show experimentally by EPR spectroscopy that the addition of 1 mM H_2_O_2_ leads to the oxidative degradation of the binuclear cluster N1b on subunit NuoG (Fig. 2). Calculations of the intramolecular electron transfer rates revealed that electron transfer from cluster N3 to N4 via cluster N1b can easily be bypassed by direct electron transfer from cluster N3 to N4, both located on NuoG (Fig. 4)^46,47^. In this case, the reduced flavin will transfer its electrons sequentially to cluster N3 as in the non-treated complex. Here, the electrons will have a sequential transient stop before they appear at clusters N4 and N5^47^. The edge-to-edge distance between N3 and N4 is 14.9 Å, which theoretically leads to an overall 10 times diminished intramolecular electron transfer rate^46,47^. This will not change the overall electron transfer rate from NADH to Q as the Q reduction and release is much slower than the intra-molecular electron transfer along the Fe/S clusters^43^. Recently, we generated a complex I variant that lacked cluster N1b by deleting the *E. coli* iron-sulfur cluster carrier protein BolA^48^. The lack of cluster N1b led to the assembly of an active complex. Importantly, the loss of N1b did not affect the NADH oxidase activity of the mutant membranes, which means that the lack of N1b did not alter the overall electron transfer rate from NADH to Q^48^.

The oxidative damage of N1b by H_2_O_2_ was unexpected as the Fe/S clusters of complex I are buried well within the protein. However, the short channel identified with the program CAVER paves the way for H_2_O_2_ from the protein surface to cluster N1b in bacterial and mitochondrial complex I (Fig. 3). Looking at the overall structure of the complex, it appears that cluster N1a on NuoE is most exposed to the aqueous medium (Fig. 3). On the other hand, the protein environment of N1a is more hydrophobic than that of the other clusters. Indeed, CAVER identified a channel with a diameter of 1.98 Å in *E. coli* and 1.9 Å in ovine complex I, respectively, (Fig. 3C,D) leading from the protein surface directly to N1a. Remarkably, however, this channel is lined with apolar atoms at the respective constrictions. Most likely, this hydrophobic surface of the channel prevents H_2_O_2_ from damaging cluster N1a as well.

Taken together, our data clearly show that physiological H_2_O_2_ concentrations do not damage the Fe/S clusters of respiratory complex I. Furthermore, elevated concentrations specifically damage cluster N1b. However, N1b damage does not affect complex I activity, as this cluster can be bypassed during intramolecular electron transfer, thus preserving the physiological activity of the complex.

## Methods

### Strains, plasmid and cell growth

A derivative of *E. coli* strain BW25113^49^, chromosomally lacking the gene *ndh*, was used as host to overproduce complex I^48^. The chromosomal *nuo*-operon of this strain was replaced by a resistance cartridge (*npt*II). The host strain was transformed with plasmid pBAD*nuo*_*His*_ encoding the entire *nuo*-operon^50^. Expression of the *nuo*-operon was induced by an addition of 0.2% (w/v) l-arabinose. For protein preparation, cells were grown at 37 °C in a rich autoinduction medium containing 34 µg/mL chloramphenicol while agitating at 180 rpm^51^. According to the experimental setup, all NADH-induced activities of membranes of the transformed strain originate from the catalytic activity of overproduced complex I encoded by the plasmid.

### Preparation of cytoplasmic membranes

Cells were harvested by centrifugation, suspended in 50 mM MES/NaOH, 50 mM NaCl, pH 6.0 (buffer A) containing 0.1 mM phenylmethylsulfonyl fluoride (PMSF) and a few grains DNAseI and disrupted by three passages through an HPL-6 (Maximator, 1000–1500 bar)^51^. Cytoplasmic membranes were obtained by differential centrifugation^51^ and suspended in an equal volume (1:1, w/v) of buffer A with 5 mM MgCl_2_ and 0.1 mM PMSF.

### Protein preparation

The complex was prepared as described^52^. In short, membrane proteins were extracted with 2% (w/v) lauryl maltose neopentyl glycol (LMNG; final concentration), the cleared extract was adjusted to 20 mM imidazole and applied to a 35 mL ProBond Ni^2+^-IDA column (Invitrogen) equilibrated in buffer A with 5 mM MgCl_2_, 10% (v/v) glycerol, 0.005% (w/v) LMNG and 20 mM imidazole at pH 6.8. Bound proteins were eluted with the same buffer containing 308 mM imidazole. Fractions containing NADH/ferricyanide oxidoreductase activity were pooled, concentrated by ultrafiltration in 100 kDa MWCO Amicon Ultra-15 centrifugal filter devices (Millipore) and polished using a Superose 6 size exclusion chromatography column (300 mL, GE Healthcare) equilibrated in buffer A with 5 mM MgCl_2_, 10% (v/v) glycerol and 0.005% (w/v) LMNG. The fractions with highest NADH/ferricyanide oxidoreductase activity were used for further studies.

### Activity assays

Activity assays were performed at 30 °C. The NADH oxidase activity of cytoplasmic membranes was determined with a Clarke-type oxygen electrode (DW1; Hansatech) as described^51^. The electrode was calibrated by adding a few grains of sodium dithionite to air saturated buffer^51^. The NADH/ferricyanide oxidoreductase activity was determined as decrease of the ferricyanide absorbance at 410 nm with a diode-array spectrometer (QS cuvette, d = 1 cm, Hellma; TIDAS II, J&M Aalen) using a ε of 1 mM^−1^ cm^−1^^53^. The assay was performed in buffer A containing 1 mM ferricyanide and 0.2 mM NADH. The reaction was started by the addition of the protein and the rate of the enzymatic reaction was corrected by the value of the non-enzymatic reaction. The NADH:decyl-Q oxidoreductase activity was measured as decrease of the NADH concentration at 340 nm using an ε of 6.3 mM^−1^ cm^−1^ (QS cuvette, d = 1 cm, Hellma; TIDAS II, J&M Aalen). Purified complex I was mixed in a 1:1 (w/w) ratio with *E. coli* polar lipids (10 mg mL^−1^; Avanti) and incubated on ice for 30 min. The assay contained 60 µM decyl-Q, 2 µg complex I and a tenfold molar excess (5 µg) *E. coli* cytochrome *bo*_*3*_ oxidase in buffer A with 5 mM MgCl_2_, 10% (v/v) glycerol and 0.005% (w/v) LMNG. The reaction was started by an addition of 150 µM NADH^46^. Different concentrations of H_2_O_2_ (30%, v/v; Chemsolute) were added to the assays just before the start of the reaction.

To determine the reversibility of H_2_O_2_ inhibition, membranes were incubated for 5 min with 20 mM H_2_O_2_. Aliquots of treated and non-treated membranes were centrifuged (178,000*g*, 4 °C, 60 min, rotor 60Ti, Sorvall wX + ultra centrifuge, Thermo Scientific) and re-suspended in the tenfold volume buffer A with 5 mM MgCl_2_ and 0.1 mM PMSF. This procedure was repeated two times and the NADH/ferricyanide and NADH oxidase activity of both samples were determined.

### EPR spectroscopy

EPR measurements were conducted with an EMX 6/1 spectrometer (Bruker) operating at X-band. The sample temperature was controlled with an ESR-9 helium flow cryostat (Oxford Instruments). Spectra were recorded at 40 K and 2 mW microwave power and at 13 K and 5 mW microwave power from 300 to 380 mT. Other EPR conditions were: microwave frequency, 9.360 GHz; modulation amplitude, 0.6 mT; time constant, 0.164 s; scan rate, 17.9 mT min^−1^. 300 µL complex I (2.5–3.5 mg mL^−1^) in buffer A were reduced with a 2000 fold molar excess NADH (10–14 mM) and shock frozen at 150 K in 2-methylbutane/methylcyclohexane (1:5; v:v).

To determine whether H_2_O_2_ oxidizes or damages cluster N1b, complex I was incubated with 1 mM H_2_O_2_ for 5 min. The excess H_2_O_2_ was removed by concentrating the sample by ultrafiltration (Amicon Ultra-15, MWCO: 100 kDa, Millipore; 3800 g, 4 °C, rotor A-4-44, centrifuge 5804R, Eppendorf) and subsequent tenfold dilution in buffer A with 5 mM MgCl_2_, 10% (v/v) glycerol and 0.005% (w/v) LMNG. This procedure was repeated two times. An EPR spectrum of the concentrated sample reduced by a 2000 fold molar excess NADH was recorded.

### Calculation of solvent channels

Solvent accessibility of the Fe/S clusters of *E. coli* and ovine complex I was probed with the PyMOL plugin CAVER (version 3.0.3)^54,55^ at minimal radii of 0.90–1.20 Å using the pdb model 7AWT of the peripheral arm containing all clusters^44^ and the pdb model 7ZD6 of ovine complex I^36^.

### Other analytical methods

Protein concentration was determined according to the biuret method using BSA as a standard^56^. The concentration of purified complex I was determined by UV/vis-spectroscopy (TIDAS II, J&M Aalen) using an ε of 781 mM^−1^ cm^−1^ as derived from the amino acid sequence^57^. SDS-PAGE (sodium dodecyl sulfate–polyacrylamide gel electrophoresis) was performed with a 10% separating gel and a 3.9% stacking gel^58^. A possible oxidation of FMN and NADH by H_2_O_2_ was assayed by LC–MS analysis. 1 mM FMN and 1 mM NADH (both from Sigma Aldrich) in buffer A were incubated with 20 mM H_2_O_2_ for 30 min at ambient temperature and then subjected to HPLC (ProntoSIL 120-3-C18; AQ plus; 1 mL, 150 × 3.0 mm) at a flow rate of 0.5 mL min^−1^ in 10% triethylammonium acetate (100 mM) and 90% water. After 1 min, a gradient from 10 to 90% acetonitrile was applied over 17 min. Eluting samples were directly analyzed by API-MS (Dionex MSQ Plus).

## Supplementary Information

Supplementary Information.

## Data Availability

The data supporting the findings of this article are available from the corresponding author upon reasonable request.
