# Supplementary material for: H2O2 selectively damages the binuclear iron-sulfur cluster N1b of respiratory complex I
Source: Sci Rep. 2023 May 11;13:7652. doi: 10.1038/s41598-023-34821-5 (PMC10175503; doi:10.1038/s41598-023-34821-5)
Supplement: Supplementary file 1 — Supplementary Information. [file 41598_2023_34821_MOESM1_ESM.docx]

Supplementary Information

**H_2_O_2_ selectively damages the binuclear iron-sulfur cluster N1b of respiratory complex I**

By

Lisa Strotmann, Caroline Harter, Tatjana Gerasimova, Kevin Ritter, Henning J. Jessen, Daniel Wohlwend, Thorsten Friedrich

**Figure S1:** Purification of complex I

**Figure S2:** EPR difference spectrum at 40 K

**Figure S3:** EPR spectrum of NADH-reduced complex I after removal of surplus H_2_O_2_

**Figure S4:** HPLC-MS analysis of H_2_O_2_ treated FMN and NADH


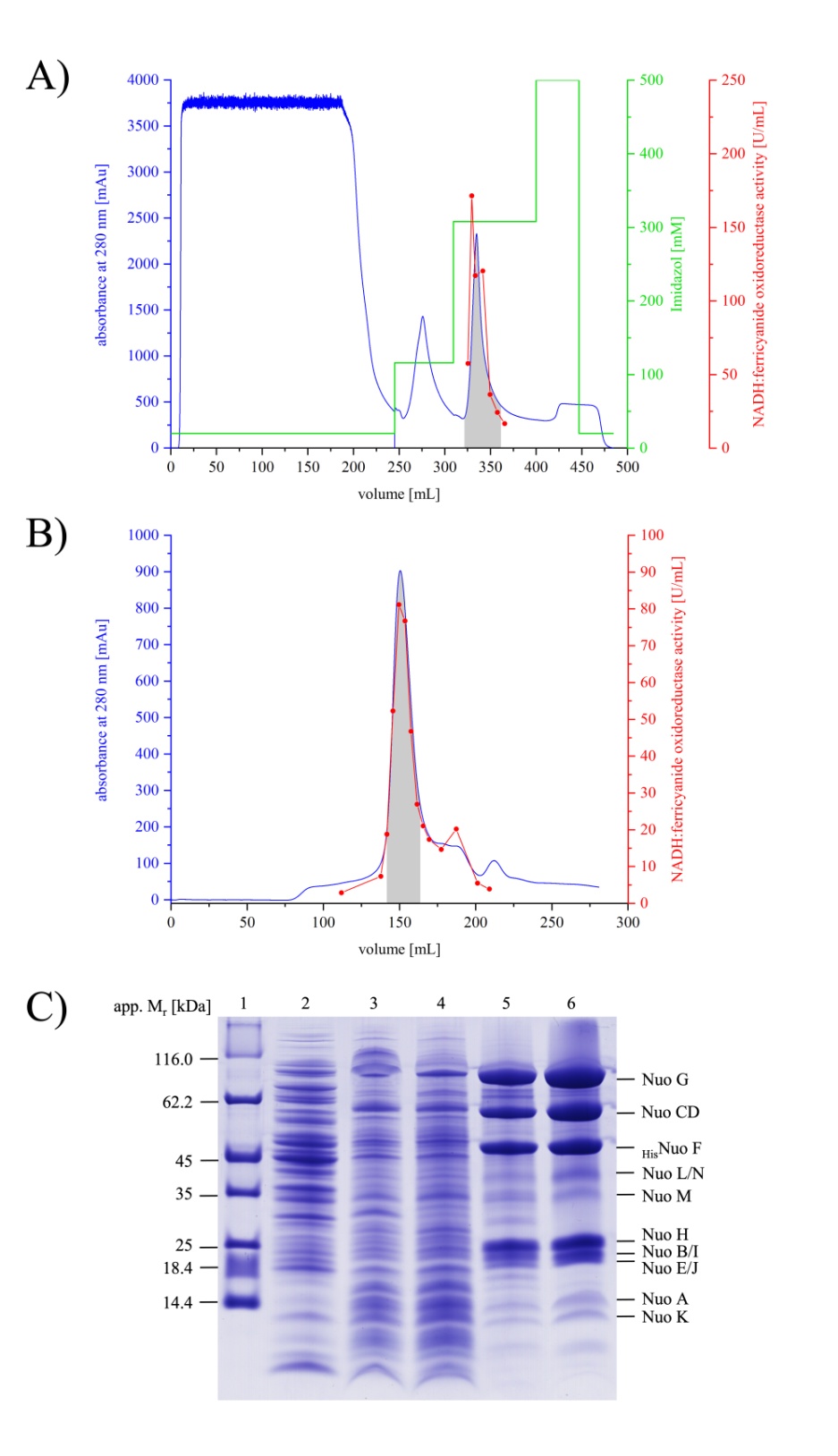


**Figure S1: Preparation of complex I from the overproducing *E. coli* strain.** Elution profiles of A) the affinity-chromatography on ProBond Ni^2+^-IDA and B) the size exclusion chromatography on Superose 6. Fractions (marked with a gray background) with highest NADH/ferricyanide oxidoreductase activity (red curve) were used in the next step. C) SDS-PAGE of a preparation of complex I. Lanes were loaded with: 1) Unstained protein marker (Thermo Scientific), 2) cytosolic fraction, 3) membrane fraction, 4) membrane extract, 5) combined fractions obtained by affinity chromatography and 6) combined fractions obtained by size exclusion chromatography. The bands are attributed to the individual subunits according to their apparent mass. The faint band at around 80 kDa represents a proteolytic digestion product of NuoG.


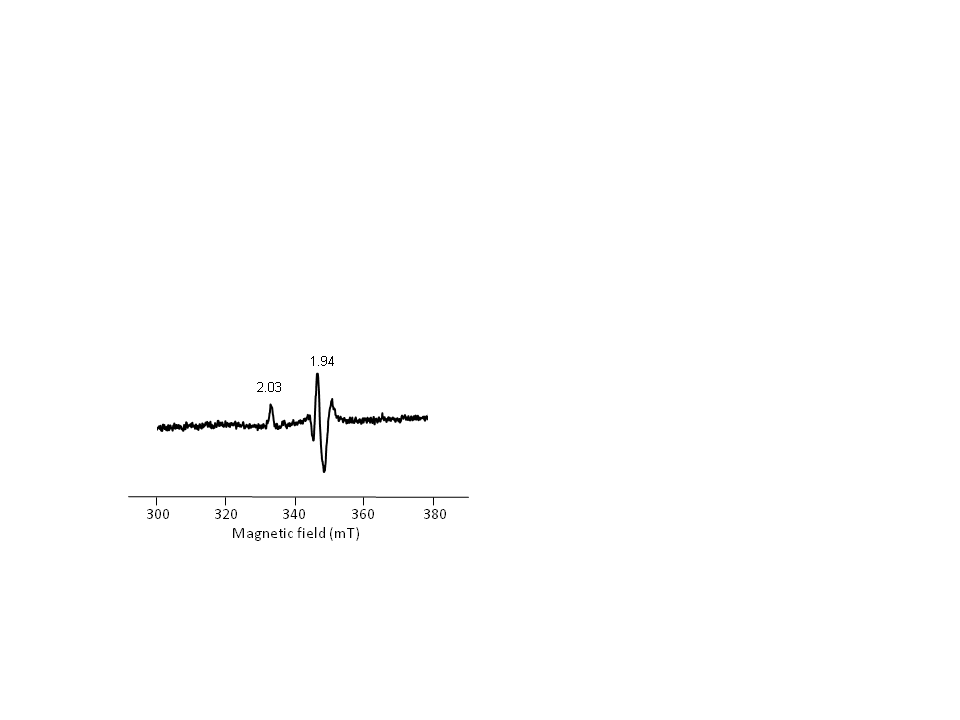


**Figure S2: EPR difference spectrum of the untreated sample minus that of the sample treated with 1 mM H_2_O_2_.** The spectra recorded at 40K and 2 mW were substracted from each other. The resulting difference displays the signals of the binuclear cluster N1b (g_//,⊥_ = 2.03 and 1.94).


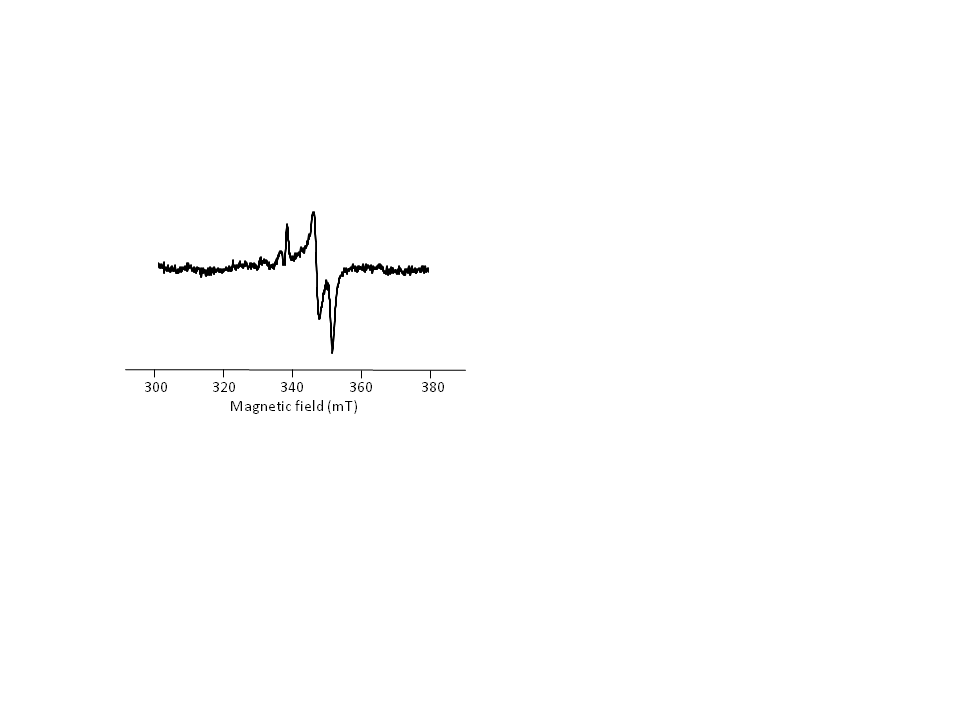


**Figure S3: EPR spectrum of NADH-reduced complex I after incubation with 1 mM H_2_O_2_ and subsequent removal of H_2_O_2_ by repeated dilution and concentration** The spectrum was recorded at 40K and 2 mW and displays the signals of the binuclear cluster N1a (g_x, y, z_ = 1.92, 1.94, and 2.00). The signal at g_z_ = 2.00 is distorted by spectral overlap with a small radical signal.


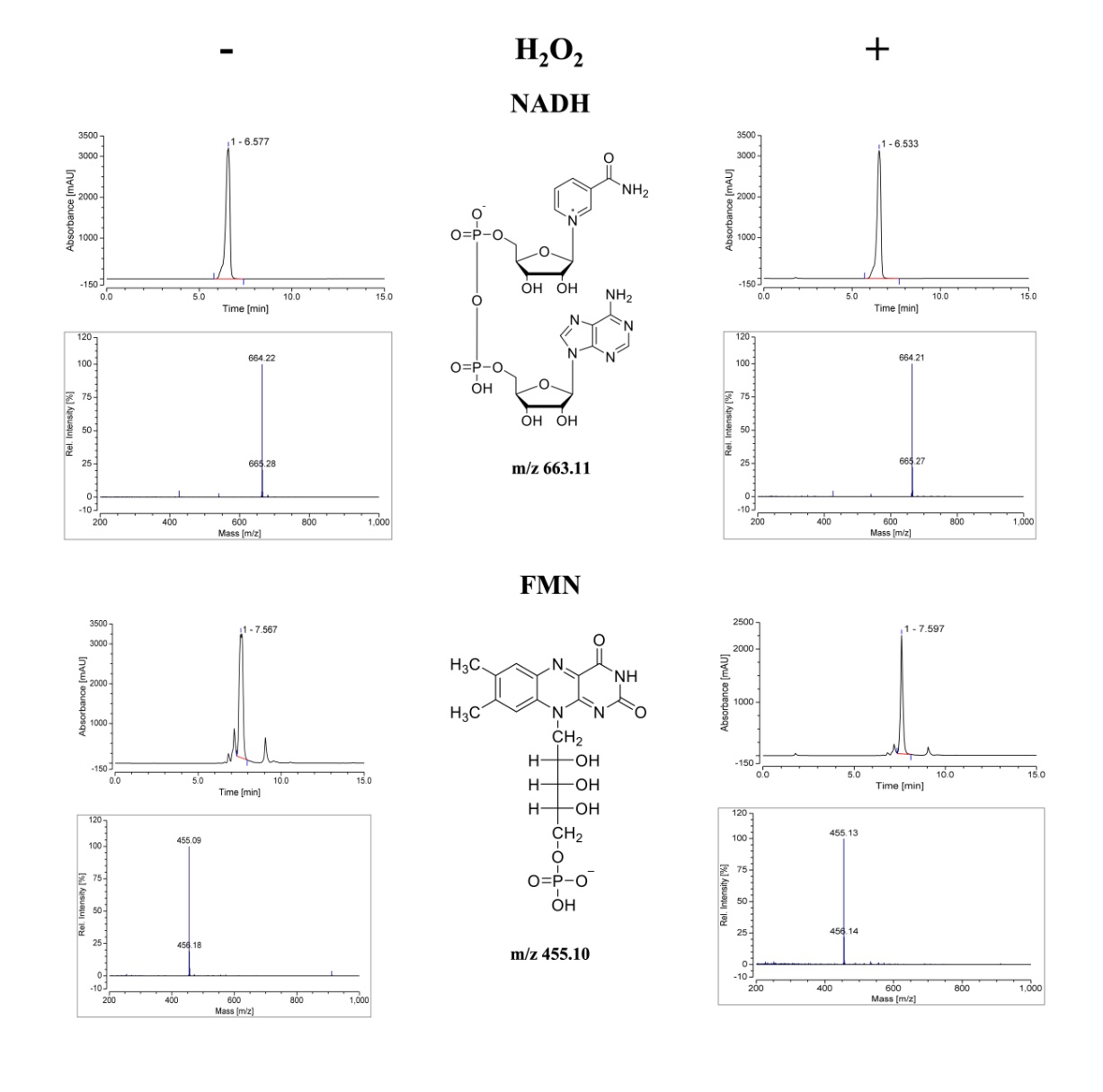


**Figure S4: 20 mM H_2_O_2_ do not oxidize NADH and FMN.** 1 mM NADH in buffer A (top) and 1 mM FMN in buffer A (bottom) were incubated with 20 mM H_2_O_2_ for 20 min at ambient temperature. HPLC-MS analysis of an untreated sample (left) and the sample incubated with H_2_O_2_ (right) revealed that the treatment did neither change the elution profile nor the molecular mass of the nucleotides.
